# Strategies to improve access to cognitive behavioral therapies for anxiety disorders: A scoping review

**DOI:** 10.1371/journal.pone.0264368

**Published:** 2022-03-01

**Authors:** Jean-Daniel Carrier, Frances Gallagher, Alain Vanasse, Pasquale Roberge

**Affiliations:** 1 Department of family medicine and emergency medicine, PRIMUS research group, Université de Sherbrooke, Sherbrooke, Canada; 2 Department of psychiatry, Université de Sherbrooke, Sherbrooke, Canada; 3 School of nursing, Université de Sherbrooke, Sherbrooke, Canada; 4 Centre de recherche du CHUS, Sherbrooke, Canada; Xiamen University - Malaysia Campus: Xiamen University - Malaysia, MALAYSIA

## Abstract

**Background:**

Strategies to improve access to evidence-based psychological treatments (EBPTs) include but are not limited to implementation strategies. No currently available framework accounts for the full scope of strategies available to allow stakeholders to improve access to EBPTs. Anxiety disorders are common and impactful mental conditions for which EBPTs, especially cognitive-behavioral therapies (CBT), are well-established yet often hard to access.

**Objective:**

Describe and classify the various strategies reported to improve access to CBT for anxiety disorders.

**Methods:**

Scoping review with a keyword search of several databases + additional grey literature documents reporting on strategies to improve access to CBT for anxiety disorders. A thematic and inductive analysis of data based on grounded theory principles was conducted using NVivo.

**Results:**

We propose to classify strategies to improve access to CBT for anxiety disorders as either "Contributing to the evidence base," "Identifying CBT delivery modalities to adopt in practice," "Building capacity for CBT delivery," "Attuning the process of access to local needs," "Engaging potential service users," or "Improving programs and policies." Each of these strategies is defined, and critical information for their operationalization is provided, including the actors that could be involved in their implementation.

**Implications:**

This scoping review highlights gaps in implementation research regarding improving access to EBPTs that should be accounted for in future studies.

## Introduction

Access to healthcare, defined as "the degree of fit between the clients and the system [1]," is a complex concept characterized by dimensions such as approachability, acceptability, availability, accommodation, affordability, and appropriateness [2]. Ever since the conceptualization of psychological interventions as well-defined treatments for specific mental health conditions, experts have highlighted the magnitude of the challenge of providing access to evidence-based psychological treatments (EBPTs) in comparison with medications [3,4]. Without the ability to rely on the well-organized supply chain of the pharmaceutical industry, improving access to evidence-based psychological treatments requires an understanding of the intervention itself, its context, and the processes leading to patient engagement in treatment [5]. This task can sometimes be facilitated when EBPTs are organized into standardized protocols delivered by digital means to facilitate their dissemination, but those innovations address only some of the existing barriers to access and do not meet the needs of every patient [6]. In the predictable future, access to EBPTs will likely still need to include human-delivered interventions to account for all the dimensions of access in meeting populations’ needs.

When interventions need to be provided by professionals, accessing them involves potential barriers not only at the intervention- and patient-levels, but also at the provider- and contextual-levels. In the last decades, the field of implementation science has been developing to help understand how to take all those potential barriers into account and allow evidence-based interventions to impact public health [7]. Implementation strategies, which are "methods or techniques used to enhance the adoption, implementation, and sustainability of a clinical program or practice" [8], have an important role in improving access to EBPTs. However, approaches to improve access to EBPTs in practice extend beyond implementation [9]. For example, innovation in treatment delivery methods should be considered among possible strategies to improve access to EBPTs even though it encroaches into the territory of treatment development [10]. Similarly, communication to address patient-level barriers to access such as stigma and misunderstanding of treatments is highly relevant to improving access to EBPTs [11], but it is distinct from implementation [9].

Anxiety disorders are highly prevalent mental conditions [12,13] characterized by anxiety, fear, and related behaviors such as avoidance [14]. Anxiety disorders have a considerable impact on quality of life and often a long-term course [15,16]. Young adults under 35 are especially affected, leading to an increased risk of suicidal behavior and of developing chronic health conditions over the lifetime [17–19]. Both pharmacological and psychological interventions are appropriate for anxiety disorders, but psychotherapy’s benefits are more likely to persist in time [20,21]. Among psychotherapies, the evidence supporting the efficacy of cognitive-behavioral therapy (CBT) for anxiety disorders is particularly consistent [22–25]. Moreover, many patients would prefer psychotherapy to medication if offered the option, sometimes strongly so [20,26–28]. This combination of evidence support and patient preferences makes CBT a prime example of an EBPT worth improving access to [29]. Consequently, actors who would be in a position to contribute to improving access to CBT for anxiety disorders need to be able to select appropriate strategies to do so, which would vary depending on their background and the characteristics of the populations and context they are involved with.

This study aims to describe and classify the various strategies reported to improve access to CBT for anxiety disorders, whether or not they were specifically designed for CBT, for anxiety disorders, or for implementation purposes.

## Methods

### Design

Given this study’s aim, we expected to find relevant information in a wide range of documents published for diverse purposes, with or without primary data, quantitative or qualitative, from clinical trials to opinion papers to clinical practice guidelines. We conducted a scoping review using Arksey & O’Malley’s framework, a flexible approach to assessing the breadth of the information available about a topic of interest [30]. We conducted this scoping review in three steps. The first step was initiated in January 2017 and included an informal literature review followed by the selection of articles and the first round of data analysis. The second step was performed in 2020 and included the integration of newly published documents and the second round of data analysis. The third step was initiated in August 2021 and consisted in the replication of the systematic scoping review methodology with updated inclusion and exclusion criteria to ensure replicability and enhance the timeliness of this paper. Except when otherwise specified, methods and results presented in this paper purport to the last step of the review.

Throughout the study, we followed the five stages of a scoping review according to Arksey & O’Malley: 1) Identifying the research question, 2) Identifying relevant studies, 3) Study selection, 4) Charting the data, and 5) Collating, summarizing, and reporting the results [30]. Reporting was guided by the PRISMA statement extension for scoping reviews [PRISMA-ScR, 31]. The PRISMA-ScR checklist is available as S1 Fig. We did not formally assess the quality of the documents included as this is not expected to be helpful in the context of a scoping review, following PRISMA-ScR. No protocol was registered for this review.

#### Stage 1: Identifying the research question

We initiated this review in January 2017 with the question: "How is CBT for anxiety disorders implemented?" We first conducted a non-systematic exploration of the literature, leading to an understanding of some perceived gaps in the current knowledge base and extension of the topic of interest beyond implementation. We then formulated the following research question: "What strategies can be used to improve access to CBT for anxiety disorders?". We did not consider post-traumatic stress disorder or obsessive-compulsive disorder among anxiety disorders as they have both been assigned their own diagnostic category by the American Psychiatric Association since 2013 [14].

### Data sources

#### Stage 2: Identifying relevant studies

The primary sources of documents were the databases Medline, Embase, CINAHL, and PsycINFO, which we accessed through the search engines Scopus, PubMed, and EBSCOhost [32]. We first selected keywords during the initial exploration of the literature and refined them by exploring the Medical Subject Headings (MeSH) classification for relevant concepts. In each database, we combined three categories of keywords using the Boolean operator "AND"; as searched in August 2021:

The target population (anxiety disorders):

["Anxiety disorder" OR "Phobia" OR "Social anxiety" OR SAD OR "Panic disorder" OR "Panic attacks" OR "Agoraphobia" OR "Generalized anxiety disorder" OR GAD].

The intervention (CBT):

["Cognitive therapy" OR "Cognitive behavioral therapy" OR CBT OR ((cognit* OR behav*) AND psychotherap*) OR "Exposure therapy"].

Possible approaches to improve access:

[Implement* OR disseminat* OR adopt* OR "Knowledge transfer" OR "Knowledge translation" OR "Knowledge exchange" OR "Research utilization" OR "Quality improvement" OR "Healthcare improvement"].

We limited the literature search to documents published in 2010 or later. We did not use language limitations in the search engines but only considered documents with English or French text available for analysis. No specific keyword was used to represent the concept of access in the search strategy; the rationale for performing this scoping review was to examine documents where the intention of improving access underlies the choice of intervention even when access is not clearly operationalized, and restricting the search to explicitly access-related documents would have defeated this purpose.

During the first steps of this review in 2017 and 2020, we also identified grey literature documents from citations in included studies and from the authors’ prior knowledge of the field to integrate policy-making and professional organizations’ perspectives. When those documents were published in 2010 or later and otherwise met the inclusion criteria of this study, we included them as additional documents.

#### Stage 3: Study selection

In the first step of this review, each abstract was independently assessed for inclusion by two authors (JDC, PR) and we calculated Cohen’s kappa at 0.47 from a sample of 200 entries for moderate inter-rater reliability. After several discussions and clarifications to the inclusion criteria, a research assistant was tasked with replicating abstracts selection in the final step of this review in 2021. From a sample of 200 entries, we calculated Cohen’s kappa at 0.94 between PR and the research assistant at this point. The research assistant then completed abstracts selection, always keeping documents that had been included in previous steps of the review. We considered a document for inclusion if it met the following criteria: 1) Includes the delivery of cognitive and/or behavioral therapy as a key component of the treatment of anxiety disorders, 2) Is relevant for adults in the general population (i.e., not a population based on pregnancy status or a specific physical condition or occupation), 3) Offers insights about at least one intervention aiming to improve access or modifiable barriers and/or facilitators of access to treatment (i.e., does not solely change the specific techniques used or the quality of treatment for individuals who already have access to CBT), and 4) Is not explicitly tailored to the distinct challenges of improving mental health services in low- or middle-income countries [33]. We considered any publication design for inclusion except for study protocols.

**Fig 1:**
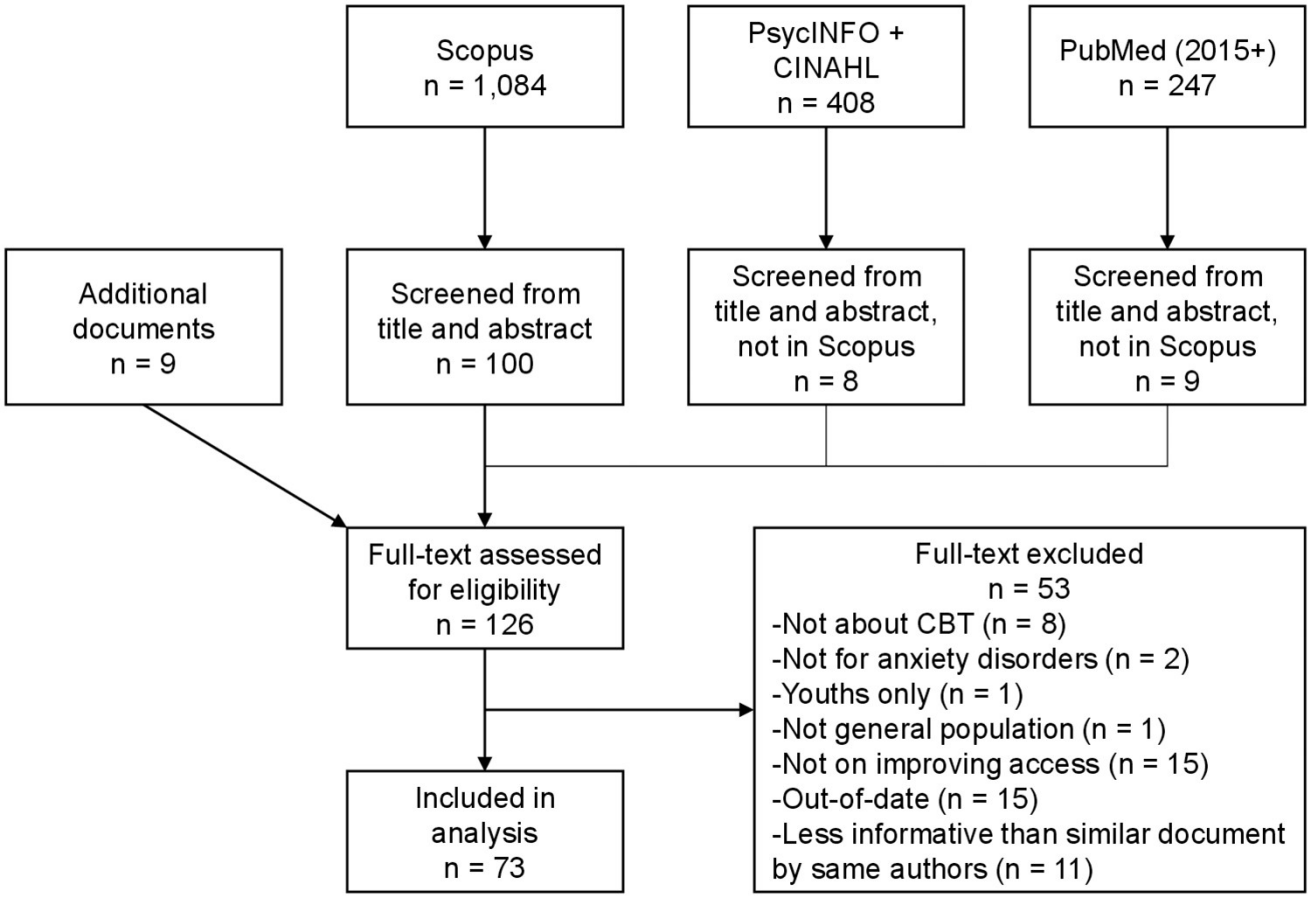
Flow diagram of this scoping review.

We attempted to retrieve electronic files for any document included by either evaluator, 25 of which could not be accessed. The first author then proceeded to assess full text using the same inclusion criteria. Notably, we excluded several empirical studies based on criterion 1 when they used an anxiety scale but did not provide information on anxiety disorder diagnostic status. To help manage redundancy between references, when two or more documents with overlapping authorship included similar content, we only included the document deemed the most informative for this review (e.g., presents more recent data or showcases a more access-oriented perspective). The decision not to analyze specific documents is presented separately from criteria-based exclusions in Fig 1.

### Data extraction and analysis

#### Stage 4: Charting the data

We extracted data using the qualitative research software NVivo 11 [34]. During the first round of analysis in 2017, we initially coded documents line-by-line to find quotes potentially relevant to the research question, which we identified as "Strategies," "Barriers," or "Facilitators." We decided to code barriers and facilitators to account for the notion that strategies are sometimes not explicitly formulated [35]. We expected that to reach this study’s aim, we would need to infer some strategies from the barriers they were intended to overcome or the facilitators they were meant to enhance. For example, some interventions were described as facing technological barriers to their implementation while others benefited from a design based on commonly used technology. Considering both of those occurrences in the documents analyzed helped us conceptualize how improving technological infrastructure could be part of a strategy building capacity for CBT delivery.

#### Stage 5: Collating, summarizing, and reporting the results

We adopted a thematic and inductive approach for data analysis following grounded theory methodology principles, including line-by-line initial coding and subsequent generation of categories through constant comparison [36]. Whenever a document was analyzed, codes were created and organized to reflect an evolving understanding of the strategies relevant to improving access to CBT for anxiety disorders. New codes were systematically compared to previous codes to detect redundancy and delineate conceptual categories. Previously analyzed documents were revisited whenever the coding structure was modified to evaluate how their content fitted into the evolving conceptual categories. This process allowed for the emergence of types and subtypes of strategies under which the various activities mentioned in the documents included could be grouped.

During the second round of analysis in 2020, we used conceptual categories previously identified as a basis for data extraction to ensure that the proposed classification was reliable in accounting for the evolving evidence base. Several clarifications to the boundaries and inner structure of those categories were possible using this new data, with theoretical saturation achieved for most strategies. While they are not implementation strategies per se, we follow Proctor et al.’s recommendations to report each strategy’s name, definition, and operationalization [8]. Throughout data analysis, several attempts were made to structure the results by comparing our conceptual categories to implementation [37], knowledge translation [38], and public mental health [39] frameworks, but it did not appear that adapting any of those models could adequately account for the full range of strategies identified. However, some existing models proved useful in validating the classification presented in this paper. Notably, we compared names and definitions of strategies with the Expert Recommendations for Implementing change [40,41], we extracted the types of actors that may be involved from Leeman et al.’s adaptation of the Interactive Systems Framework [9,42], and the main dimensions of access from Penchansky & Thomas’ model [1,2].

When we systematically replicated the scoping review methodology in 2021, we integrated documents on specific phobias which we had initially excluded and added the corresponding keyword. Additionally, we added "exposure therapy" in the keyword search, which we had not initially done, and elicited several additional documents that met our inclusion criteria. Consequently, we remained sensitive to any modification to our classification that would be warranted based on new data or perspectives, but no substantial change appeared to be justified.

## Results

**Fig 2:**
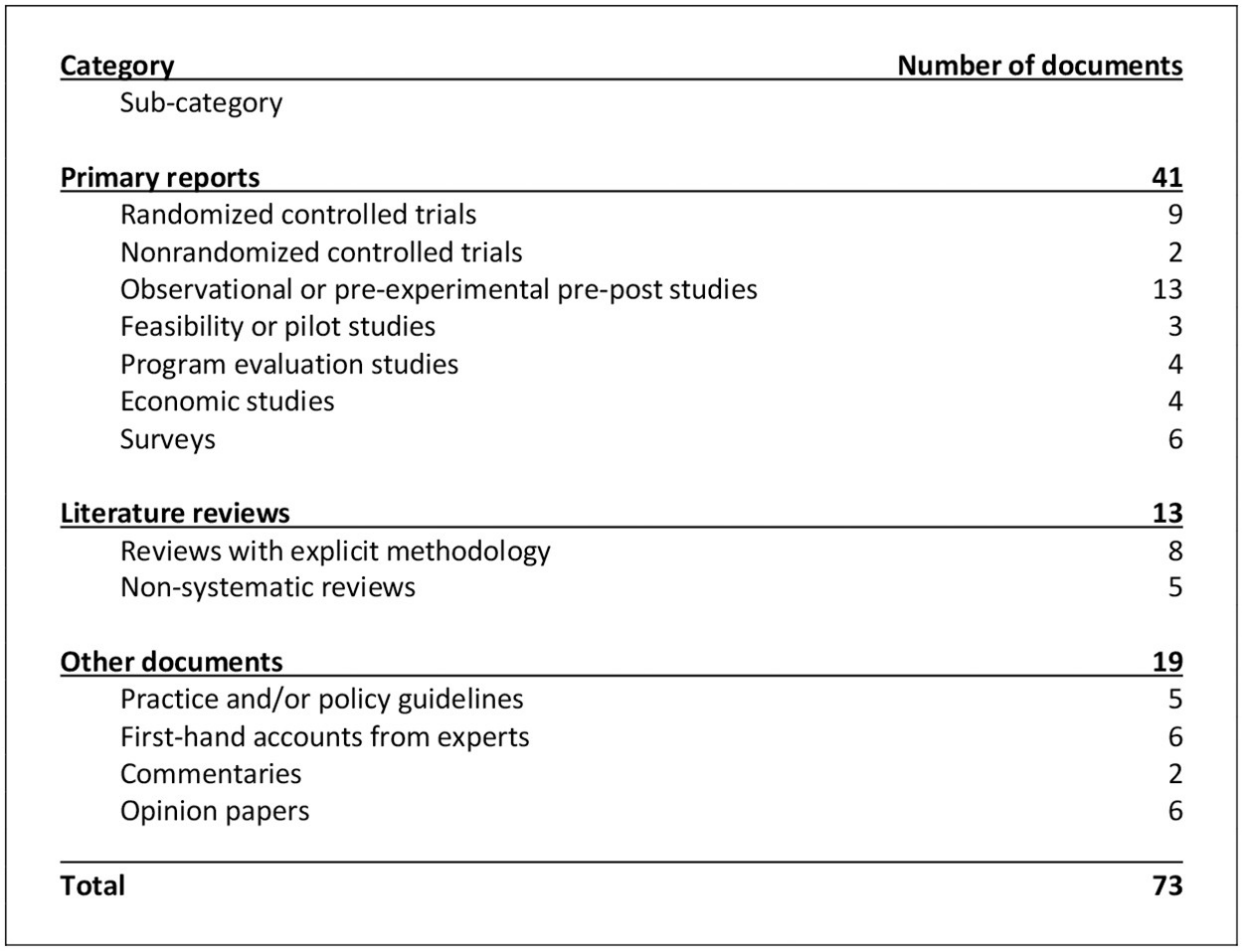
Number of documents included by publication design.

As shown in the flow diagram, we assessed the full text of a total of 215 documents, 111 of which were excluded (Fig 1). From the 104 remaining documents, 20 were not coded for analysis to limit redundancy based on similar content with overlapping authorship. A total of 10 additional documents met inclusion criteria and were integrated into the review. Among the 94 documents analyzed, 52 (55%) were empirical studies, but there was considerable variation in individual research or publication designs (Fig 2). The content of each document is summarized in S1 Table.

We identified six types of strategies to improve access to CBT for anxiety disorders: contributing to the evidence base, identifying CBT delivery modalities to adopt in practice, building capacity for CBT delivery, attuning the process of access to local needs, engaging potential service users, and improving programs and policies. The name, definition, and operationalization of each type of strategy are presented in Table 1. In this paper, we refer most often to individuals living with anxiety disorders as "patients," but readers should consider other terms such as study participants or clients to be interchangeable if they better fit their perspective and context.

**Table 1:** Types of strategies to improve access to CBT for anxiety disorders and their characteristics.

Type of strategies	Definition	Targets	Actors	Subtypes
**Contributing to the evidence base**	Participating in the generation of scientific knowledge through activities designed or adapted to bridge the gap between research and practice	Any dimensions of access	Treatment developers, researchers, research sponsors, or decision-makers involved in existing programs (independent of their direct involvement in treatment delivery)	Demonstrate specific treatments’ applicability Generate data from existing programs Conduct research targeted on access-related issues
**Identifying CBT delivery modalities to adopt in practice**	Replacing or augmenting existing services with CBT delivery modalities tailored to avoid access-related barriers	Acceptability, availability, accommodation, and affordability of treatments	Delivery system actors, including clinicians and managers	Offer remote CBT delivery Provide lower-intensity CBT interventions Adopt transdiagnostic CBT protocols
**Building capacity for CBT delivery**	Ensuring that treatment providers have the skills and resources to make evidence-based CBT a part of routine care	Availability and appropriateness of treatments	Support system actors	Train CBT providers Offer continuing support to CBT providers
**Attuning the process of access to local needs**	Clarifying and improving how various stakeholders work together to make engaging in CBT for anxiety disorders as seamless as possible for local populations	Approachability, acceptability, availability, accommodation, and affordability of treatments	Primary and secondary care clinicians and managers (independent of their direct involvement in CBT delivery), with the collaboration of synthesis and translation systems actors	Improve access mechanisms and criteria Map the process of access to locally available EBPTs Develop stakeholder interrelationships
**Engaging potential service users**	Promoting treatment-seeking through direct channels of communication with populations not yet engaged in treatment	Approachability and acceptability of treatments	Various actors with a public health mandate (independent of their direct involvement in CBT delivery)	Disseminate relevant information Increase perceived acceptability and motivation to engage in CBT
**Improving programs and policies**	Developing and disseminating frameworks to guide actions toward strategic actions to improve access to CBT for anxiety disorders	Any dimensions of access	Support system actors, with the collaboration of synthesis and translation systems actors	Develop and disseminate access-oriented standards Design scalable programs

### Contributing to the evidence base

Treatment developers, researchers, research sponsors, and decision-makers can improve access to CBT by contributing to the evidence base. For example, those actors can use the research process to **demonstrate specific treatments’ applicability** to the settings where usual care is delivered. To facilitate this process, treatment developers can ensure that front-line clinicians are involved in developing and evaluating treatment protocols [43–46]. When treatment efficacy is known, researchers can contribute further by conducting "effectiveness" [47–50] or "pragmatic" [51] studies, with treatment providers, resources, and patients representative of "real-world" contexts [11,43,52–54]. As an illustration of this approach, some authors invite researchers to be mindful of trials’ exclusion criteria that would lead clinicians to have reasonable doubts about the applicability of the results to their clientele [44,55–59]. Because of resource discrepancies, interventions trialed exclusively during experiments [44,57,60,61] or in the context of a specialized clinic [48,55,62,63] may need to be tested for transferability to build a case for dissemination. Providing a more detailed account of crucial information, such as the specific training and supervision procedures employed [51,64], can facilitate replication. Detailed reporting can also enable translation or cultural adaptation of interventions to other contexts to circumvent the need to repeat the whole treatment development process [62,65], but the effectiveness of the intervention or its adaptations still needs to be rigorously tested in different contexts [66–68]. Early involvement of partners with experience in dissemination and commercialization can help ensure that important issues are not neglected [69].

Several methodological approaches were identified to **generate data from existing programs**: naturalistic, open, or "phase 4" studies [11,44,45,62,70–72], observational studies from routine care [73], implementation or program evaluation studies [49,74–78], and quality improvement research [79]. While the degree of overlap between these methodologies is beyond this review’s scope, their variety in published studies highlights the recognized importance of learning from existing programs. In the future, some technology-based interventions may even allow for the seamless integration of randomization in addition to data collection within routine intervention delivery [80]. Documents analyzed included reports on existing programs aiming to improve access to CBT for anxiety disorders through treatment modalities such as telephone CBT [74], internet-delivered CBT [70–73,75,77,78], and transdiagnostic group CBT. Some studies reported on an implemented training program [76], an approach to collaborative care [79], or CBT practices in primary care settings [44]. Other existing programs had the broader aim of improving access to various mental health services (including CBT) through a national-level endeavor [81,82].

Researchers have a wide range of options to **conduct research targeted on access-related issues**, two of which clearly emerged from data analysis. Firstly, research on more parsimonious interventions can facilitate implementation while maintaining treatment integrity in clinical practice [83,84]. This can be achieved either by conducting separate trials for simplified versions or components of evidence-based interventions or isolating the effects of components of a complex intervention through data analysis techniques [83,84]. Research on the minimal amount of resources required to achieve treatment outcomes (e.g., complexity of the necessary exposure techniques, number and duration of sessions, amount of work expected from patients between sessions, degree of therapist involvement, therapists’ expertise level and amount of training) is also relevant to facilitate the development of low-intensity CBT interventions for integration in routine care [47,69,80,85–88]. Secondly, evidence relevant to predicting individual patient response to specific CBT delivery modalities can be gathered to allow for more informed resource allocation [46,49,73,89]. Clinically, patient response predictors can translate into automated, self-directed, or standardized assessment protocols [29,48,70,90] or in screening procedures using clinical or administrative data to identify potential treatment candidates [29,54]. This strategy also includes research on the early detection of non-response and "stepping-up" procedures to timely direct patients toward more intensive treatment modalities [63,73,88].

### Identifying CBT delivery modalities to adopt in practice

Actors involved in treatment delivery can identify CBT delivery modalities to adopt in practice that would enable them to contribute to improving access to treatment. For example, CBT providers may **offer remote CBT delivery** to circumvent several common individual-level barriers to access. Some of those barriers are logistical, such as lack of professional resources in one’s immediate environment and difficulty to afford transportation or to spend time away from professional or parental responsibilities [11,47,57,61,68,69,73–75,77,78,91–98]. Logistical issues can also originate from or be amplified by larger-scale events, of which the COVID-19 pandemic is an unavoidable example [96]. Individuals who would otherwise avoid therapy by fear of stigma may also find remote treatment acceptable [49,52,69,73,74,91,92,98,99]. Remote treatment can also facilitate access for patients who face symptoms inherent to anxiety disorders such as agoraphobia or social anxiety [52,61,67,75,92,96,100,101]. In the documents included, remote CBT was sometimes mainly delivered in real-time by telephone, videoconferencing, or real-time chat [73,74,94–98]. In other cases, remote CBT was provided using either self-guided treatment alone [47,52,91], self-guided treatment augmented with asynchronous interactions with a coach or therapist [57,67,70–72,75,77,100], or a blend of self-guided treatment and real-time interactions with a therapist [49,61,65,101]. While we present remote CBT delivery as a singular strategy, it is notable that CBT delivery modalities are commonly blended in practice. Indeed, most self-guided treatment modalities (discussed below) were also accessible remotely.

Treatment providers can also **provide lower-intensity CBT interventions** such as self-guided treatment, which allows for a higher number of patients to be treated without increasing the number of therapists needed. Self-guided CBT, either provided alone or as part of a blended treatment, can potentially reduce the time therapists need to allocate to each patient [24,54,58,61,62,65,68,77,86,90,91,93,99,101,102]. While self-guided CBT may not be a good fit for everyone [103], some patients would prefer to attempt managing their symptoms by themselves before seeing a therapist face to face [58,104]. Clinical guidelines acknowledge that self-guided treatment can be accessed using physical tools such as books or manuals [24,105], but computerized self-guided CBT figured more prominently in the documents included. Computerized CBT protocols let patients interact with a computer program that never deviates from the intended treatment and can be easily updated to account for the evolving evidence base [48,59,61]. While computerized CBT can be delivered in a dedicated physical setting, it is typically offered as internet-delivered CBT (iCBT) and accessible through any device connected to the internet, with some exceptions requiring a mobile device [52,69,100]. Even if they might not replace face-to-face therapy, self-guided treatments can benefit patients on waiting lists [58,99] or be used as part of a stepped-care treatment delivery model [24,49,63,81,92,106,107]. Although there are exceptions and the field is rapidly evolving [80], virtual reality interventions are not typically self-guided. However, virtual reality can help provide in vivo exposure when it would require therapists to manage highly complex or resource-intensive situations, which may be unrealistic in clinical practice [43,50,69,80,89,108–110]. In situations where its effectiveness has been demonstrated, virtual reality has often been found to be more acceptable to patients than in vivo exposure [43,50,89,102,110]. Group therapy can also be used as a lower-intensity CBT delivery modality, decreasing therapist time required per patient. Some of the documents included attested to the feasibility and effectiveness of conducting group CBT adaptations [24,96,103,111], including in primary care settings. Group therapy’s benefits were also often leveraged through a transdiagnostic approach to treatment (discussed below). Lower-intensity CBT interventions also include brief or modular CBT protocols designed to save time for both patients and therapists while maintaining a significant degree of treatment efficacy [83,86,87,112,113].

CBT providers can **adopt transdiagnostic CBT protocols** (tCBT) to target common symptoms and processes across anxiety disorders or even between anxiety disorders and other mental health conditions [11,49,52,53,55,62,111,114,115]. Accordingly, tCBT competency enables therapists to provide CBT for a higher proportion of their patients with the same amount of training [11,49,51,53,55,83,111,114,115]. The transdiagnostic approach also facilitates treatment delivery for the numerous patients with comorbidities encountered in clinical settings [11,53,104,115,116]. Offering transdiagnostic programs for CBT delivery can also facilitate implementation and mitigate the clinical challenge of finding out where to send patients with less common conditions or presentations [45]. Therefore, although the economic advantages of tCBT are yet to be empirically demonstrated, transdiagnostic protocols are promising options to limit the costs of CBT when delivered either as a group therapy modality [51,55,111] or through websites or mobile applications [49,52,116].

### Building capacity for CBT delivery

Support system actors can build capacity for CBT delivery to improve access to CBT, first and foremost by **training CBT providers**. On the one hand, clinical psychologists and other therapists should be adequately trained in CBT and other EBPTs when graduating [45,46,76,117,118]. On the other hand, training clinicians to deliver CBT when they are already in a clinical position can be invaluable to improve local capacity, especially in primary care or for underserved locations and populations [56,66,68,79,113,119]. For active practitioners, mitigating the direct and indirect training costs is crucial to getting them involved [83,85], notably through online training programs [91,117–120]. In some contexts, training non-psychotherapist staff to deliver CBT components can help compensate for the lack of specialized human resources [48,64,79,81,83,113,121,122]. The specific training required to attain competency in CBT delivery depends on both the treatment modality and the trainee’s skills and previous experience [46,83,123]. Whatever their background, several authors suggest that clinical supervision is critical to prepare CBT providers for practice [11,44,63,85,117,118,124]. Training should be congruent with the role trainees are expected to fulfill on the field, covering new delivery modalities such as remote CBT and self-guided treatments [69,71,74,81,82,92,98] and the specifics of managing in vivo or virtual reality exposure in a clinical setting [43,89,125]. Some wide-spread misconceptions about CBT can also be addressed in training to encourage its integration in practice. Those misconceptions include, for example, that exposure is generally harmful or unacceptable to patients [43,45,54,118,119,123,126] or that patients who do not meet inclusion criteria of published clinical trials should be excluded from access [45,54].

**Offering continuing support to CBT providers** on the field may be vital in ensuring that they practice evidence-based CBT [45,113,117,119,123]. Indeed, programs developed to provide EBPTs can quickly evolve toward general counseling services unless considerable efforts are put forward to maintain treatment integrity [81,98], starting with explicit directives and support from mental healthcare organizations to train in and adopt CBT and EBPTs [11,44,117,123,125]. Additional support may be offered through motivational enhancement [119] or ongoing clinical feedback or supervision after completing training [64,79,117–119,125,127]. Online tools and learning communities can also improve trainees’ ability to provide evidence-based CBT in practice [45,117,119]. If clinicians are to implement CBT, support system actors can ensure that they have access to the necessary resources, such as treatment manuals [82] or technological infrastructure [50,68,89,96,97]. Providing support for outcomes monitoring was described as an opportunity for mutually beneficial long-term collaborations between the fields of academia, management, and treatment delivery [44,57,60,78,81]. While systematic outcomes monitoring is standard in clinical trials, real-world CBT providers often need support to routinely collect data even when they have a favorable opinion of such practices [79,127].

### Attuning the process of access to local needs

Actors involved in decision-making have an important role in attuning the process of access to local needs, including by **improving access mechanisms and criteria** on which reaching CBT is conditional. Mechanisms of access include payment models, as anxiety disorders patients who must pay out-of-pocket may decide to forgo receiving CBT even when it is readily available. Meanwhile, offering CBT at little or no charge has often been suggested to be cost-effective or even profitable from the perspectives of a public healthcare system [66,76,78,85,113,128,129], public insurers covering private services [11,69,71,113,127–129], and private insurers [45,69,94,128]. Access to CBT for anxiety disorders can also be improved by promoting early detection mechanisms [90,105] and educational efforts to acquaint clinicians with the CBT delivery modalities available in their context [62,74,113]. It is also often possible to eliminate mandatory additional steps delaying treatment, including specialist evaluation [70] and physician prescription requirement [29,74,105,127]. Allowing patient self-referral is often viable [77,78,81] but should not be the only available access path [82,127]. When deciding on administrative criteria to qualify for treatment, focusing on eligibility rather than exclusion criteria can mitigate the risk of depriving a substantial proportion of anxiety disorders patients of what often remains their best option [59]. For patients living with a handicap affecting communication, assessment should use adapted tools and/or involve caregivers as required [29]. For patients belonging to a language or cultural minority, clinicians should be aware of the proper mechanisms to reach bilingual and culturally sensitive therapists [29], to procure the assistance of a translator [29], or to steer patients toward specialized self-help resources [69].

**Mapping the process of access to locally available EBPTs** for anxiety disorders can be instrumental in bridging the gap between the evidence base and patient needs. Stakeholders sometimes have very different ideas about the best course of action to reach treatment providers, potentially leading to some confusion [98,127]. Mapping the process of access from a local-population perspective can reduce the level of confusion by making explicit the respective mandate of each category of stakeholders involved in providing access to CBT [29,81,105]. Specifically, an organizational device called "local care pathways" has been promoted, which involves modeling of the various "pathways" a patient could use to access EBPTs whomever they encountered first in the mental healthcare system [29]. To maximize their usefulness, local care pathways may be constructed by a coalition of primary and secondary care clinicians, managers, and other local stakeholders [29]. Only locally available interventions are included in local care pathways, organizing EBPTs in a stepped-care fashion. In stepped-care models, the least intensive interventions that are reasonably likely to result in clinical improvement are provided first, allowing mental health organizations to maximize the cost-effectiveness of treatments when providing for large populations [24,29,63,81,82,103,105–107,113,129,130]. The evidence-based design of a locally applicable stepped-care model including CBT for anxiety disorders is expected to be increasingly important as further knowledge is gathered about the consequences of changing the order and timing at which specific interventions are provided [47,106,130].

The process of access can also be attuned to local needs by **developing stakeholders’ interrelationships**. At the heart of such collaborations, primary care mental health services are expected to oversee most of the population’s needs and treatments for common mental disorders [11,29,113]. Especially relevant in primary care, collaborative care models can be adopted to improve the chances that a given patient receives appropriate care at the right time [79]. Collaborative care models generally involve a care manager and promote collaborative approaches such a co-location and sharing a common electronic files system [60,113,128,129]. Collaboration between primary care clinicians and anxiety disorders specialists such as psychiatrists or clinical psychologists is sometimes required to clarify the need for EBPTs [105,128]. Making the transition seamless between primary care and specialist care enables specialist evaluation to improve access to treatment rather than simply delay it [105]. In access-oriented collaborations, specialists can play a supervisory role regarding primary care clinicians’ capacity to deliver CBT [64] and eventually fill in for temporary gaps in the CBT services located in specific primary care practices. Treatment providers should have some form of contractual relationship with primary care stakeholders [121], who can also pool their resources to afford expensive technological infrastructure or physical locations for group therapy [94]. Stakeholders’ interrelationships can also involve outside organizations mandated to detect and refer people who potentially need CBT for anxiety disorders, such as employment support agencies and non-profit organizations. Some organizations can also help make CBT more accessible by providing childcare services or travel assistance to eligible patients [29].

### Engaging potential service users

Various actors can contribute to improving access to CBT for anxiety disorders by engaging potential service users. **Disseminating relevant information** can help individuals with anxiety disorders in the community adopt the behaviors required to reach CBT services. Indeed, literacy and knowledge about anxiety disorders and their possible treatments are the first steps in procuring EBPTs for oneself and can be improved by direct communication [11,120,124,131]. Awareness campaigns in clinicians’ waiting rooms, through information websites, or using social or traditional media can be considered [60,90,123,124,132], with some authors advocating highly targeted direct-to-consumer marketing for specific forms of psychotherapy [43,120,123,124]. While possibly controversial, direct-to-consumer marketing may lead to consultations enabling clinicians to detect anxiety disorders and convince patients to try CBT even when they were initially reluctant [124]. Anxiety disorders experts can make themselves periodically available for questions to provide reliable information to the public, either in person or through social media [124]. Online clinics, mobile self-help applications, and brief psychoeducation interventions can all have the dual purpose of CBT delivery and providing information to individuals independently of their immediate need or readiness to engage in psychotherapy [77,105,124,131]. Given that the best ways to reach different population segments vary, opportunities to collaborate with communication specialists should not be overlooked [124].

For some people, information about anxiety disorders and their treatments will not translate into treatment-seeking behaviors unless it also **increases their perceived acceptability and motivation to engage in CBT**. Several authors suggest that general efforts should be undertaken to reduce stigma regarding mental health issues and their treatment [11,120,123,124,131,133]. Pervasive ideas to address include that mental disorders equate to moral failure or that people living with anxiety ought to cope with their symptoms by themselves [11,133]. While education can contribute to reducing stigma [105], opportunities should also be created for members of the public to try out and see CBT modalities in action [124]. Indeed, observability and trialability are known factors impacting the acceptability of engaging in an intervention [99]. For some patients, for example those suffering from severe agoraphobia, initiating CBT at home may be the only acceptable option [122]. In other cases, the option to engage in CBT in primary care rather than specialist settings may be enough to improve acceptability [60]. Even when treatment appears acceptable, patients who worry more tend to perceive more barriers to engage in treatment [133]. Perceived barriers sometimes have to be addressed directly with individuals, and preparatory psychoeducative or motivational interventions have been suggested as potential tools to increase anxiety disorders patients’ willingness to engage in CBT [11,56,103,126,131]. Independently of the specific techniques that may be used to convince patients, individuals experiencing a supportive therapeutic relationship with a clinician are more likely to be open to trying an intervention despite initial resistance [58,103].

### Improving programs and policies

Improving programs and policies is the last type of strategy that we identified in this review. Actors involved in policymaking can **develop and disseminate access-oriented standards** to create expectations that individuals should follow throughout the mental healthcare system. Standards regarding access to CBT for anxiety disorders can be related to the other types of strategies identified in this review (Table 1). Standards contributing to the evidence base include rules for publicly financed research to consider the dissemination of interventions in real-world situations [132]. Standards identifying CBT delivery modalities to adopt in practice include the homologation of EBPTs by an authoritative body at the national level [11,69,80,120,121]. Regulations on new treatment delivery modalities such as iCBT can also be access-oriented as long as they take their particular nature into account and avoid making them prohibitively expensive to develop or impossible to access between jurisdictions [61,69,94]. Standards building capacity for CBT delivery may address psychotherapists’ training and mandate that they have been exposed to CBT for anxiety disorders before graduating [45,46,66]. They can also set continuing education criteria to maintain a license to practice specific CBT delivery modalities or CBT in general [69]. Standards attuning the process of access to local needs were diverse in the documents included. Policymakers may alleviate costs to patients through regulations requiring or incentivizing CBT coverage by private insurance companies [11,45,113,129] or gearing public coverage of healthcare services toward evidence-based guidelines [68,73,76,81,107,120,128,129]. Benchmarks for the expected timing of access to CBT [105] and guidance on integrating specific CBT delivery modalities in routine care [92,130] clearly communicate access-oriented standards to local actors, as do regulations either defining the scope of practice of various service providers or clarifying the optimal staff composition of clinical teams providing CBT [129]. Finally, standards on potential service user engagement can be established by regulating the advertisement of psychological treatments [124] and providing official channels to help the public validate the credibility of various sources of information, especially on the internet [69,90,124].

**Designing scalable programs** is a significant challenge. Some national programs such as Improving Access to Psychological Therapies (IAPT) in the UK [73,81,130] and Better Access in Australia [74] have received a considerable amount of attention in the documents included. Two broad principles to improve the scalability of programs could be highlighted from this review. First, proposed program implementation activities should be coherent with local organizations’ resources and prior practices [11,78,97,132]. Indeed, taking clinicians’ current skills and practice into account helps reduce the cost and acceptability of implementation [60,66,85,129]. Program designers should also build-in enough clarity and flexibility to allow program adaptation to local needs without unnecessary administrative burden for clinicians and managers [60,74,121,129,132]. Rolling-out a program gradually can be considered, focusing first on those organizations with capabilities most closely matching the program’s design [81]. Program implementers can also encourage testing various program versions and prioritize disseminating the most successful [69,129]. Second, program developers should strive to allocate resources through mechanisms acceptable to most stakeholders, including clinicians [49,60,85,127,128] and local managers [121]. Communication about the proposed changes’ potential advantages should be clear [74] and accompanied by due process to take stakeholders’ interests into account. Stakeholders’ engagement may start with building a strong case for the cost-effectiveness of providing CBT for anxiety disorders in a given context [60,85,107] and developing a shared vision for a program that would prove politically defensible [81]. Buy-in can be facilitated by comparison with other successful programs [44,76,82,127]. Program design may also improve long-term access by anticipating and preventing funds’ reallocation to other priorities over time by local authorities [81].

## Discussion

This scoping review presents a comprehensive overview of the various strategies reported to improve access to CBT for anxiety disorders in the extant literature. We used a combination of search keywords that were general enough in scope to allow for the retrieval of documents arguing in favor of one or several strategies even when the authors had not explicitly conceptualized access as an outcome. Our approach to data analysis, based on grounded theory principles, enabled us to construct a classification closely fitting the current state of empirical knowledge on improving access to EBPTs. Notably, we were able to identify, define, and operationalize types of strategies to improve access to CBT for anxiety disorders (Table 1) from activities described in published documents rather than restricting our findings to an existing conceptual framework that would be only partially applicable.

Some of the documents included proposed categorizations that were influential in pushing our analysis forward. Harvey & Gumport used a five-level model to discuss modifiable barriers to access for EBPTs and possible solutions to address them: "patient-level," "therapist-level," "treatment-level," "organization-level," and "government-level" [11]. Gunter & Whittal suggested that dissemination of CBT for anxiety disorders is impeded by specific and general barriers that could be addressed with a sequential dissemination model: "accumulation of an evidence base," "obtaining support via an appeal to clinical practice guidelines and cost-effectiveness," "training and implementation," and "ongoing research and validation" [45]. Cartreine et al.’s "Roadmap to Computer-Based Psychotherapy" includes research milestones, training milestones, policy milestones, and industry milestones [69]. However, none of those articles report the methodology used to reach their proposed classification. In the field of implementation science, publications from the ERIC study showcase a rigorous approach to classifying implementation strategies [40,41], which contributed to the final label and definition of some of the strategies presented in this review. Despite this contribution, implementation strategies would not be expected to encompass the whole scope of strategies to improve access to CBT for anxiety disorders. Indeed, Leeman et al. argue that within the field of implementation science, "implementation processes" should be distinguished from "dissemination," "integration," "capacity-building," and "scale-up strategies" [9]. Even from this broader perspective, some of the strategies identified in this review would still be unaccounted for, most significantly "contributing to the evidence base" and "engaging potential service users.

The issue of improving access to EBPTs highlights how implementation and public health perspectives may overlap in real-world contexts. Considering the application of existing implementation frameworks to an access-related public health issue (i.e., inequities in healthcare delivery), Baumann & Cabassa faced a dilemma similar to ours [134]. Consequently, they propose to "reframe" several elements of implementation frameworks to facilitate their application to healthcare inequities research, touching on several strategies identified in our review when they suggest, for example, to "focus on reach from the very beginning," "develop the science of adaptation," or "use an equity lens for implementation outcomes" [134]. In that respect, our study is complementary in approaching this question from the other direction. Through definition and classification of strategies reported to improve access to CBT for anxiety disorders, we provide a framework focused on the public health issue of access that should nonetheless feel familiar and useable to implementation experts.

Some limitations of this study should be noted. First, although the selection of documents involved an independent assessment of abstracts by two researchers, some judgment calls were necessary given the open-ended formulation of the research question. Even though Cohen’s kappa was high at 0.94, this was the result of repeated discussions among the research team rather than the inherent precision of the inclusion criteria. Therefore, while all the data used here are published and accessible, replication by another team would likely lead to a somewhat different selection of documents. Other researchers might also have given different weight to some content of the documents included, leading to another way to organize the concepts constructed through analysis. Among the strategies identified, it seems unlikely that those that repeatedly emerged from various sources–i.e., for which we reached data saturation–would have been significantly affected by selecting a different sample of documents or attributing a different level of importance to some content. However, we may have missed some promising strategies accounted for in few published documents. Second, this review remains mostly focused on articles published in peer-reviewed journals as they proved sufficient to complete data analysis. While we included some grey literature, their number is limited and biased toward our own situation in Canada as they allowed us to better understand the practical significance of various strategies through juxtaposition with familiar issues from our healthcare system. Therefore, we invite researchers interested in specific strategies in a context differing significantly from our own to delve deeper into the relevant grey literature. Third, our methods led us to get acquainted with documents presenting a rationale linking specific actions with improving access to CBT for anxiety disorders. For many of the strategies and concepts discussed in this review, we may not cite the most authoritative references that would be familiar to content experts, but rather the work of authors who have approached them with a perspective fitting our research question.

## Conclusions

This paper is one of the first to conceptualize strategies aiming to improve access to EBPTs from a systematic assessment of published documents. While each strategy can be pursued through various interventions, most programs employ more than one strategy. It seems likely that program designers are aware of the multidimensional challenges of improving access, and that they innovate by integrating complementary strategies into a single package. Unfortunately, the information reaching local actors might make access-improving programs appear more like "one size fits all" than the careful combination of complementary strategies. Building on our results, tools could be developed to facilitate local actors’ decision-making in implementing programs to better fit patient needs. For example, types and subtypes of strategies could be used to structure the content of organizational guidelines and facilitate the engagement of actors who may usually struggle to envision how their actions contribute to improving access to EBPTs for anxiety disorders. Furthermore, practice guidelines targeting a specific category of actors such as CBT providers or primary care clinicians could expand their scope through the development of add-ons on interventions to improve access to EBPTs through strategies that would be congruent with the guidelines’ users’ situation. Ultimately, using a framework of the potential strategies to improve access to EBPTs might deliver some of the answers needed to fill the remaining gaps between generating evidence, developing policies and programs, and providing access.

## Supporting information

S1 Fig Preferred Reporting Items for Systematic reviews and Meta-Analyses extension for Scoping Reviews (PRISMA-ScR) checklist. (TIF)

S1 Table Description of the documents included (n = 94), by publication design. (DOCX)
