# Correction: Strategies to improve access to cognitive behavioral therapies for anxiety disorders: A scoping review

**DOI:** 10.1371/journal.pone.0314222

**Published:** 2024-11-14

**Authors:** Jean-Daniel Carrier, Frances Gallagher, Alain Vanasse, Pasquale Roberge

Figs 1 and 2 are uploaded incorrectly. Please see the correct Figs 1 and 2 here.

**Fig 1:**
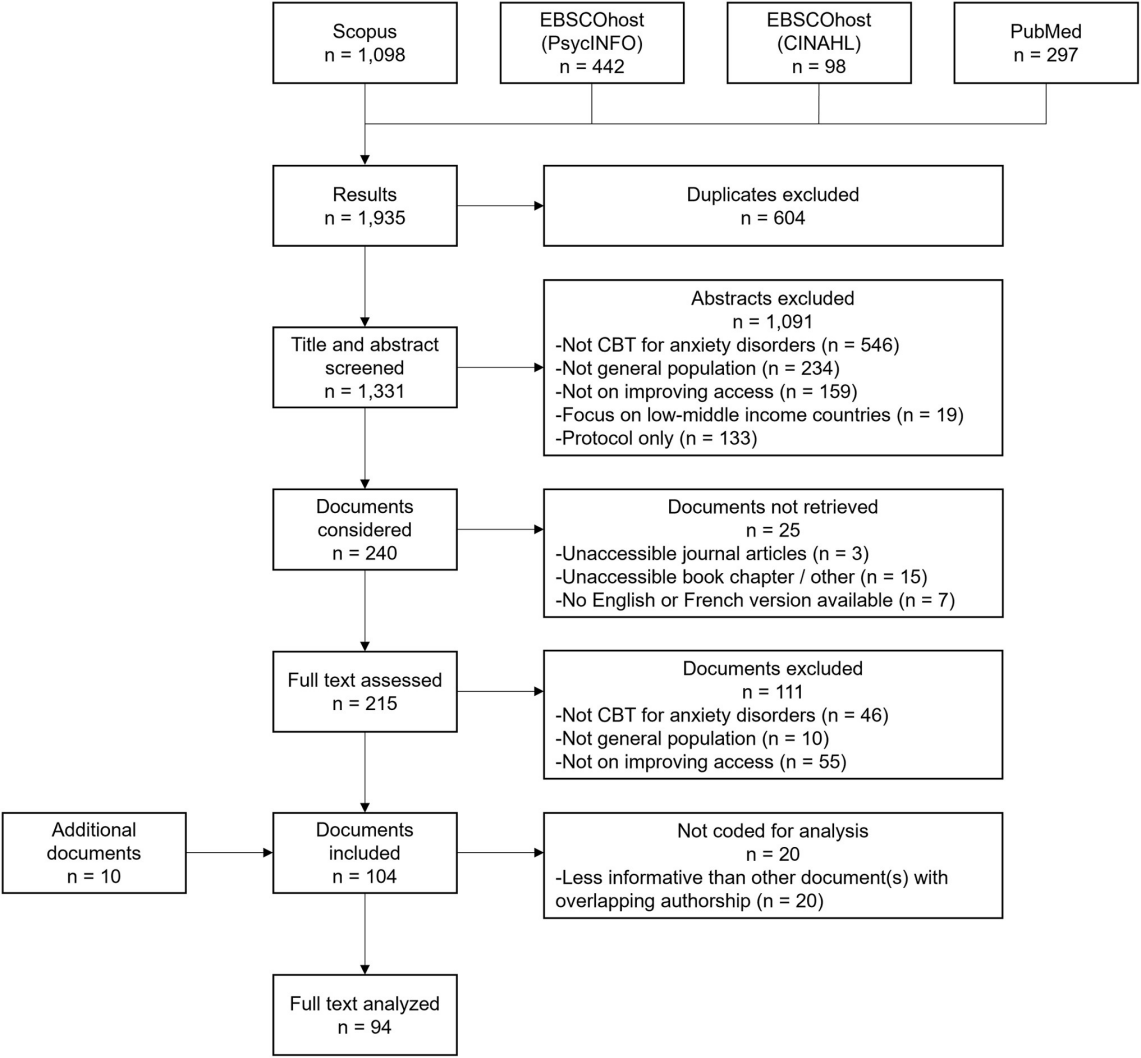
Flow diagram of this scoping review.

**Fig 2:**
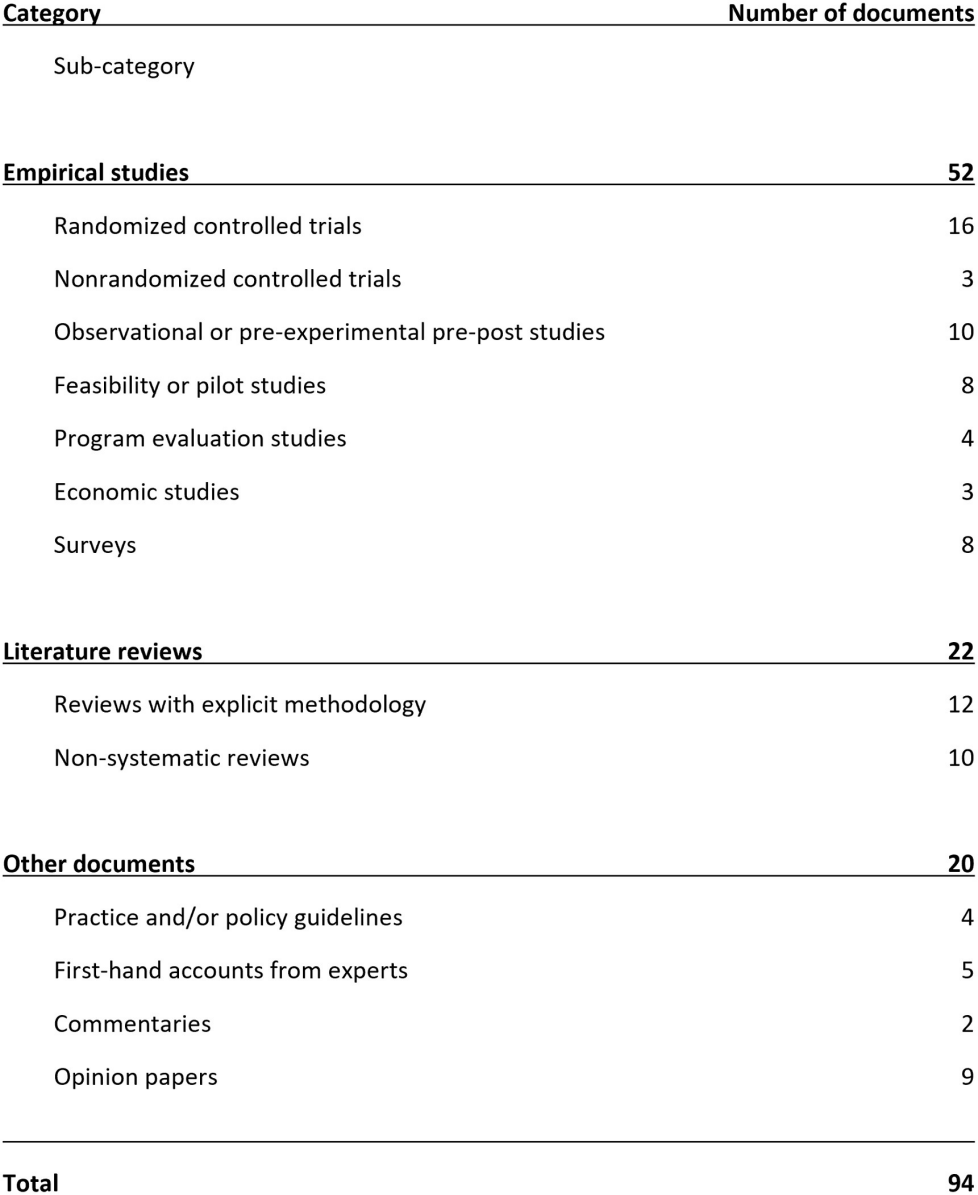
Number of documents included by publication design.
