# Supplementary figures and images for: Strategies to improve access to cognitive behavioral therapies for anxiety disorders: A scoping review
Source: PLoS One. 2022 Mar 1;17(3):e0264368. doi: 10.1371/journal.pone.0264368 (PMC8887746; doi:10.1371/journal.pone.0264368)

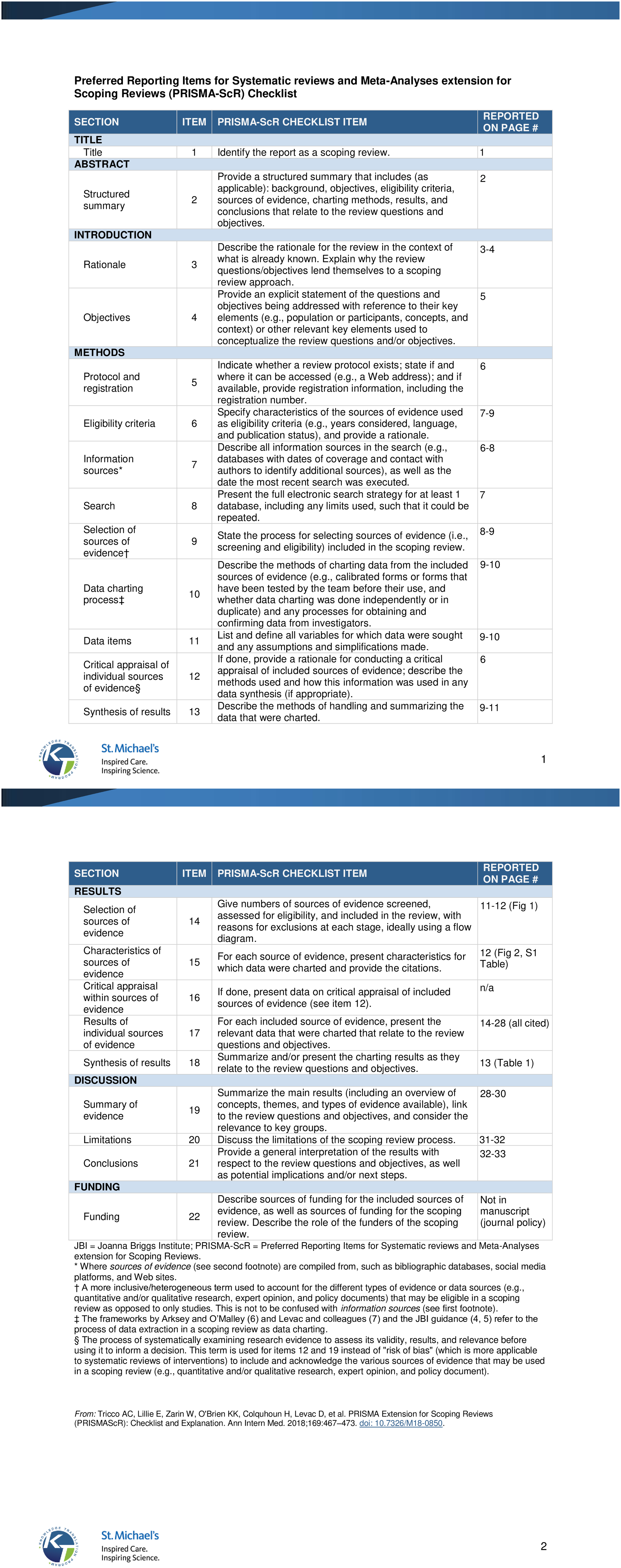

Supplement: S1 Fig — (TIF) [file pone.0264368.s001.tif]
