# Supplementary material for: Strategies to improve access to cognitive behavioral therapies for anxiety disorders: A scoping review
Source: PLoS One. 2022 Mar 1;17(3):e0264368. doi: 10.1371/journal.pone.0264368 (PMC8887746; doi:10.1371/journal.pone.0264368)
Supplement: S1 Table — (DOCX) [file pone.0264368.s002.docx]

**S1 Table. Description of the documents included (n = 94), by publication design.**

| **Empirical studies (n = 52)** | | | | | | | | | | | |
| --- | --- | --- | --- | --- | --- | --- | --- | --- | --- | --- | --- |
| **Reference** | **Document type** | **Intervention** | **Clinical target** | **Setting** | **Data source** | **Types of strategies** | | | | | |
|  |  |  |  |  |  | **Contributing to the evidence base** | **Identifying CBT delivery modalities** | **Building capacity for CBT delivery** | **Attuning the process of access** | **Engaging potential service users** | **Improving programs and policies** |
| **Randomized controlled trials (n = 16)** | | | | | | | | | | | |
| Amir et al., 2019 | Peer-reviewed journal article | Home-based computerized attention bias modification and applied relaxation vs clinical monitoring | Generalized anxiety disorder | Research clinic in San Diego, CA, USA | Self-reports + clinician assessments (n = 109) | X | X |  | X |  |  |
| Barlow et al., 2017 | Peer-reviewed journal article | Transdiagnostic CBT vs diagnosis-specific CBT vs waiting list | Anxiety disorders + obsessive-compulsive disorder | Specialized treatment center in Boston, MA, USA | Clinician assessments (n = 223 patients) | X | X |  |  |  |  |
| Botella et al., 2010 | Peer-reviewed journal article | Internet self-administered vs face-to-face CBT for fear of public speaking vs waiting list | Social anxiety disorder | Recruited via advertisement on two university campuses, Valencia, Spain | Clinician assessments (n = 127) |  | X | X |  |  |  |
| Christoforou et al., 2017 | Peer-reviewed journal article | Diagnosis specific mobile application vs general mobile application for anxiety | Agoraphobia | Patients recruited via digital advertising, UK | Self-reports (n = 170) | X | X |  |  |  |  |
| Gershkovich, 2015 | Academic paper | Acceptance-based iCBT vs iCBT + minimal therapist support | Social anxiety disorder | Patients recruited via digital advertising and referrals, USA | Clinician assessments + self-reports (n = 42) | X | X |  |  |  | X |
| González-Robles et al., 2020 | Peer-reviewed journal article | Transdiagnostic internet-delivered CBT vs usual specialist care | Anxiety disorders + others | Public specialized mental health care services in three hospitals, Spain | Clinician assessments + self-reports (n = 200) | X | X |  |  |  | X |
| Harned et al., 2014 | Peer-reviewed journal article | Online training (OT) vs OT + motivational enhancement (ME) vs OT + ME + Web-based learning community | Exposure for anxiety disorders | Practicing clinicians from varied backgrounds in Seattle, WA, USA | Self-reports (n = 181 clinicians) |  |  | X |  |  |  |
| Kampman et al., 2020 | Peer-reviewed journal article | Stepped care (self-help followed by manualized CBT) vs regular CBT | Panic disorder | Specialized mental healthcare institutions, Netherlands | Clinician assessments + self-reports (n = 128) |  | X |  | X |  |  |
| Kraus-Schuman et al., 2015 | Peer-reviewed journal article | CBT by bachelor-level vs PhD-level therapists | Generalized anxiety disorder in older adults | Patients referred from general and geriatric clinics in Houston, TX, USA | Self-reports + independent audiotape ratings (n = 147 patients, 242 CBT sessions) | X |  | X |  |  |  |
| Landy, 2017 | Academic paper | Brief psychoeducation (BP) vs BP + motivation-based session | Social anxiety disorder | Patients recruited through an online service, USA | Self-reports (n = 268) |  |  |  |  | X |  |
| Nordgreen et al., 2016 | Peer-reviewed journal article | Stepped-care vs direct face to face CBT | Social anxiety disorder + panic disorder | Public specialty mental health outpatient  clinics, Western Norway | Clinician assessments + self-reports (n = 173) | X | X | X | X |  |  |
| Norton & Barrera, 2012 | Peer-reviewed journal article | Transdiagnostic group CBT vs diagnosis-specific group CBT | Anxiety disorders | University of Houston anxiety disorders clinic, Houston, TX, USA | Clinician assessments + self-reports (n = 46) |  | X |  |  |  |  |
| Roberge et al., 2020 | Peer-reviewed journal article | Transdiagnostic group CBT delivered in community-based care settings + usual care vs usual care alone | Anxiety disorders | Recruited through an advertisement campaign in three regions, QC, Canada | Clinician assessments + self-reports (n = 231) | X | X |  |  |  |  |
| Stolz et al., 2018 | Peer-reviewed journal article | Guided iCBT on smartphone vs on personal computer vs waiting list | Social anxiety disorder | Recruited through an advertisement campaign, Switzerland | Self-reports (n = 150) |  | X |  |  |  |  |
| Stubbings et al., 2013 | Peer-reviewed journal article | Videconference-based vs face to face CBT | Anxiety disorders + others | University psychology clinic in Curtin, Australia | Clinician assessments + self-reports (n = 26) |  | X | X |  |  | X |
| Tulbure et al., 2015 | Peer-reviewed journal article | Guided iCBT vs waiting list | Social anxiety disorder | Recruited though an advertisement campaign, Romania | Self-reports (n = 76) | X | X |  |  |  |  |
| **Non randomized controlled trials (n = 3)** | | | | | | | | | | | |
| Boyd et al., 2019 | Peer-reviewed journal article | Implementation of a "stratified" vs "progressive" approach to stepped-care | Anxiety disorders + depression + others | Patients referred to IAPT program in one site, North-East England, UK | Self-reports (n = 4,291 stratified, 5,145 progressive) |  |  |  | X |  | X |
| Catarino et al., 2018 | Peer-reviewed journal article | Internet-enabled CBT vs severity-matched cohort receiving unspecified IAPT services | Anxiety disorders + depression + others | Self-referred or referred by a primary healthcare worker in four regions of England, UK | Clinician assessments + self-reports (n = 1,725) | X | X |  |  |  | X |
| Mason & Andrews, 2014 | Peer-reviewed journal article | Accessing iCBT through automated assessment vs specialist assessment | Anxiety disorders + depression | Patients referred by physicians to a specialized iCBT clinic, NSW, Australia | Self-reports (n = 173 automated, 135 specialist assessment) | X | X |  | X |  |  |
| **Observational or pre-experimental pre-post studies (n = 10)** | | | | | | | | | | | |
| Bogucki et al., 2021 | Peer-reviewed journal article | Short-term CBT in a primary care setting | Anxiety disorders + OCD + PTSD | Referred to a Mayo Clinic's program from 11 primary care clinics, MN + WI + AZ, USA | Clinician assessments (n = 1,589) | X |  | X |  |  | X |
| Dryman et al., 2017 | Peer-reviewed journal article | Open-access iCBT with optional coach support | Social anxiety disorder symptoms | Patients referred by therapists and recruited via digital and other media, USA | Self-reports (n = 1,683) | X | X |  |  |  |  |
| Hadjistavropoulos et al., 2014 | Peer-reviewed journal articles | Therapist-assisted iCBT in regular clinical practice | Symptoms of generalized anxiety disorder, panic disorder or depression | Patients recruited via digital and other media and referrals from primary care, SK, Canada | Self-reports (n = 221) | X | X | X |  |  |  |
| Knapstad et al., 2018 | Peer-reviewed journal article | Implementation of a national program to improve access to EBPTs | Anxiety disorders + depression symptoms | Patients referred by professionals or self-referred in twelve pilot sites, Norway | Self-reports (n = 928) | X |  | X | X |  | X |
| Kobak et al., 2017 | Peer-reviewed journal article | Technology-enhanced training protocol on CBT including didactic training, applied training and implementation in usual care | CBT for anxiety disorders + obsessive-compulsive disorder + post-traumatic stress disorder | Clinicians recruited through national professional organizations, USA | Self-reports + knowledge tests + independent audiotape ratings + patient outcome measures (n = 70 trainees, 25 with patient outcome measures) |  |  | X |  |  |  |
| Lindner et al., 2021 | Peer-reviewed journal article | One-session virtual reality exposure therapy in a routine care setting | Public speaking anxiety (all participants met social anxiety disorder criteria) | Private psychologist clinics specializing in CBT, Sweden | Clinician evaluations + self-reports (n = 20) | X | X | X |  |  |  |
| Nordgreen et al., 2018a | Peer-reviewed journal article | Guided iCBT | Panic disorder | Patients referred by general practitioners to a specialized iCBT clinic in Bergen, Norway | Self-reports (n = 114) | X | X | X | X |  |  |
| Nordgreen et al., 2018b | Peer-reviewed journal article | Guided iCBT | Social anxiety disorder | Patients referred by general practitioners to a specialized iCBT clinic in Bergen, Norway | Self-reports (n = 145) | X | X |  |  |  |  |
| Raeder et al., 2020 | Peer-reviewed journal article | Virtual-reality delivered exposure therapy | Specific phobias | Mental health research and treatment center, Germany | Self-reports + clinician evaluations + biological measures (n = 53) | X |  |  |  |  |  |
| Titov et al., 2017 | Peer-reviewed journal article | iCBT accessed through an online clinic | Anxiety + depression + obsessive-compulsive + post-traumatic stress symptoms | Patients self-referred to the MindSpot online clinic, Australia | Self-reports (n = 4,064) | X | X |  | X | X |  |
| **Feasibility or pilot studies (n = 8)** | | | | | | | | | | | |
| Croft & Hackmann, 2013 | Peer-reviewed journal article | Home-delivered CBT by trained volunteers vs psychologist-delivered CBT | Agoraphobia with a history of panic disorder | Referred by primary or secondary care clinicians in Oxford, UK | Self-reports (n = 70) |  |  | X |  | X |  |
| Kobori et al., 2014 | Peer-reviewed journal article | CBT delivered by trainees with previous clinical experience | Social anxiety disorder + obsessive-compulsive disorder + bulimia nervosa | Outpatient clinic at Chiba university hospital in Chiba, Japan | Feedback from trainees + self-reports from patients (n = 22 trainees, 43 patients) | X |  | X | X |  | X |
| Kraepelien et al., 2019 | Peer-reviewed journal article | Clinical routine to determine whether transdiagnostic or diagnosis-specific CBT would be offered | Panic disorder + social anxiety disorder + depression + insomnia | Internet psychiatry clinic based in Stockholm, Sweden | Program data + self-reports (n = 16 patients assessed using new routine, 13 old routine) |  | X |  |  |  |  |
| Mitsopoulou et al., 2021 | Peer-reviewed journal article | Single-session behavior therapy followed by self-treatment with telephone support if needed | Panic disorder | Specialized behavior therapy unit in Athens, Greece | Clinician evaluations + self-reports (n = 11) | X | X |  |  |  |  |
| Nauphal et al., 2021 | Peer-reviewed journal article | Teleconference-delivered group CBT | Social anxiety disorder | Specialized outpatient mental health clinic in Boston, MA, USA | Clinician evaluations + self-reports (n = 5) |  | X | X |  |  |  |
| Pinjarkar et al., 2015 | Peer-reviewed journal article | Brief CBT (6 sessions) | Social anxiety disorder | Outpatient psychiatric service in Bangalore, India | Clinician evaluations + self-reports (n = 7) |  | X |  |  |  |  |
| Stott et al., 2013 | Peer-reviewed journal article | Guided internet-delivered cognitive therapy | Social anxiety disorder | Anxiety disorders clinic, UK | Self-reports (n = 11) |  | X |  |  |  |  |
| Thew et al., 2019 | Peer-reviewed journal article | Therapist training in iCBT | Social anxiety disorder | Training of experienced therapists from local clinical services, Hong Kong | Trainee data + patient self-reports (n = 3 trainees, 6 patients) | X | X |  |  |  |  |
| **Program evaluation studies (n = 4)** | | | | | | | | | | | |
| Bassilios et al., 2014 | Peer-reviewed journal article | Telephone-based EBPTs implemented in usual care | Anxiety disorders + depression + others | Nation-wide services for patients referred by general practitioners, Australia | Program data + qualitative interviews with managers and clinicians + patient data (n = 22 managers, 10 clinicians, 908 patients) | X | X | X | X |  | X |
| Curran et al., 2012 | Peer-reviewed journal article | Collaborative care model with anxiety clinical specialists delivering CBT vs usual care | Generalized anxiety disorder + panic disorder + social anxiety disorder + PTSD | Seventeen internal or family medicine clinics, WA + CA + AR, USA | Qualitative interviews (n = 14 anxiety clinical specialists, 18 physicians, 13 nurses, 8 clinic administrators, 8 other clinic staff) | X |  | X | X | X | X |
| Pedersen et al., 2020 | Peer-reviewed journal article | Internet-based self-directed CBT program | Panic disorder + social anxiety disorder | Randomized controlled trial in a specialized outpatient clinic, Denmark | Semi-structured qualitative interviews (n = 12) | X | X |  |  | X |  |
| Williams et al., 2018 | Peer-reviewed journal article | Implementation of an evidence-based services delivery model in usual care | Anxiety disorders | Mayo primary care clinic in Rochester, MN, USA | Qualitative data on implementation + patient data (n = 57 patients) | X |  | X | X |  |  |
| **Economic studies (n = 3)** | | | | | | | | | | | |
| Crome et al., 2017 | Peer-reviewed journal article | Training of sufficient duration to become competent in CBT | Potential costs for social anxiety disorder | Psychologists in private practice, Australia | Previous systematic review on the duration of training + public data on direct and indirect costs | X |  | X | X |  | X |
| Matsumoto et al., 2020 | Peer-reviewed journal article | Videoconference-delivered CBT | Cost-effectiveness for panic disorder + social anxiety disorder + OCD | Recruited from a feasibility study, Japan | Self-reports (n = 25) + published financial data |  | X |  |  |  |  |
| Stiles et al., 2019 | Peer-reviewed journal article | Stepped-care including self-help and face-to-face CBT vs care as usual | Cost-effectiveness for anxiety disorders | National healthcare system, Australia | Literature review on treatment effectiveness + Australian epidemiological data |  | X |  | X |  | X |
| **Surveys (n = 8)** | | | | | | | | | | | |
| Becker-Haimes et al., 2020 | Peer-reviewed journal article | In vivo exposure therapy | Organizational conditions that may be uniquely necessary to support exposure | Specialists from top 5 most populous cities, USA + clinicians who participated in an exposure therapy workshop from the region of Philadelphia, PA, USA | Online survey (n = 24 clinical leaders in specialty clinics, 19 community clinicians) |  |  | X |  |  |  |
| Bradley & Drapeau, 2014 | Peer-reviewed journal article | Potential components of a government-funded psychotherapy program | Attitudes of clinicians for anxiety disorders + others | French-speaking licensed psychologists and psychotherapists in active practice, QC, Canada | Survey (n = 1,275 clinicians) |  |  | X | X |  | X |
| Brantnell et al., 2020 | Peer-reviewed journal article | Internet-administered CBT | Barriers and facilitators to implementation | Decision-makers in primary care organizations, Sweden | Online survey (n = 404 decision-makers) |  |  | X | X |  | X |
| Brenes et al., 2015 | Peer-reviewed journal article | Seeking mental health treatment in rural communities | Barriers perceived by older adults with generalized anxiety disorder | Older adults in rural communities, NC, USA | Mailed survey (n = 478) |  |  |  |  | X |  |
| Carper et al., 2013 | Peer-reviewed journal article | Computer-based delivery of psychological treatments | Perceptions of patient and clinician for anxiety disorders + depression | Patients and clinicians in a specialized clinic in Boston, MA, USA | Online survey (n = 55 patients, 26 clinicians) |  | X |  |  | X |  |
| Gros et al., 2013 | Peer-reviewed journal article | Delivering evidence-based psychotherapy through videoconference | Anxiety disorders + other diagnoses | Telehealth experts, USA | Online survey + literature review using keyword search (n = 8 experts) |  | X |  | X |  | X |
| Meyer et al., 2014 | Peer-reviewed journal article | Decision to provide exposure therapy for an individual patient | Justifications to exclude patients from exposure therapy for anxiety | Clinicians with experience in exposure therapy for anxious clients identified through various therapist directories, USA | Survey (n = 182) |  |  | X |  | X |  |
| Story, 2014 | Academic paper | Use of CBT by nurse practitioners | Knowledge about CBT and perceived barriers to its use for anxiety + depression | Family practice and geriatrics nurse practitioners participating to a professional meeting, AZ, USA | Pencil and paper survey (n = 20) |  | X | X | X |  | X |
| **Literature reviews (n = 22)** | | | | | | | | | | | |
| **Reference** | **Document type** | **Intervention** | **Clinical target** | **Setting** | **Data source** | **Types of strategies** | | | | | |
|  |  |  |  |  |  | **Contributing to the evidence base** | **Identifying CBT delivery modalities** | **Building capacity for CBT delivery** | **Attuning the process of access** | **Engaging potential service users** | **Improving programs and policies** |
| **Reviews with explicit methodology (n = 12)** | | | | | | | | | | | |
| Apolinário-Hagen, 2019 | Peer-reviewed journal article | Efficacy and acceptability of internet-delivered treatments | Panic disorder | Randomized controlled trials in any setting | Narrative review with keyword search (n = 8 documents included) |  | X |  |  |  |  |
| Efron & Wootton, 2021 | Peer-reviewed journal article | Efficacy of remote CBT | Panic disorder | Controlled trials in any setting | Systematic review (n = 21 documents) |  | X |  |  |  |  |
| Kandasamy & Campbell, 2019 | Policy document | Perspectives on psychotherapy (including CBT) and pharmacotherapy | Generalized anxiety disorder + depression | Qualitative studies in settings comparable to the Canadian healthcare model | Systematic review (n = 29 studies; 20 on CBT, 11 on generalized anxiety disorder) |  | X |  | X | X |  |
| INESSS, 2015 | Policy document | Modalities and conditions for access and coverage of psychotherapy | Anxiety disorders + depression + others | National healthcare system, QC, Canada | Narrative review with keyword search + grey literature |  |  |  | X |  | X |
| Maples-Keller et al., 2017 | Peer-reviewed journal article | Using virtual reality technology therapeutically, including for exposure therapy | Anxiety disorders + others | Not specified | Narrative review with keyword search | X | X | X |  |  |  |
| McManus et al., 2010 | Peer-reviewed journal article | Transdiagnostic EBPTs | Rationale for use in anxiety disorders | Not specified | Keyword search + author search (n = 17 documents included) | X | X |  |  |  |  |
| Meurk et al., 2016 | Peer-reviewed journal article | Electronic mental health services | Policy relevance for anxiety disorders + depression | National healthcare system, Australia | Systematic review (n = 30 documents included) |  |  |  |  | X | X |
| Oing & Prescott, 2018 | Peer-reviewed journal article | Virtual reality treatments | Anxiety disorders + others | Peer-reviewed publications in any setting | Systematic review (n = 49 documents included) |  | X |  |  |  |  |
| Tuerk et al., 2018 | Peer-reviewed journal article | Clinical videoconferencing to deliver EBPTs | Anxiety + depression | Not specified | Narrative review with keyword search |  | X | X | X |  |  |
| Wade, 2010 | Peer-reviewed journal article | Using the internet to assist in treatment (information, screening, therapy) | Anxiety + depression as primary conditions | Clinical trials, meta-analyses, randomized controlled trials, and reviews | Narrative review with keyword search | X | X |  | X | X | X |
| Wechsler et al., 2019 | Peer-reviewed journal article | Virtual reality vs in vivo exposure therapy | Specific phobias + agoraphobia + social anxiety disorder | RCTs in any setting | Systematic review with meta-analysis + qualitative review (n = 9 studies, 371 patients) |  | X |  |  |  |  |
| Wilks et al., 2016 | Peer-reviewed journal article | Relationship between exclusion rates and criteria and treatment outcomes in iCBT | Anxiety disorders + depression | Randomized controlled trials in any setting | Systematic review with multidimensional meta-analysis (n = 26 documents included) | X | X |  | X |  |  |
| **Non-systematic reviews (n = 10)** | | | | | | | | | | | |
| Andersson & Hedman, 2013 | Peer-reviewed journal article | Implementation and testing of guided iCBT in clinical settings | Anxiety disorders + others | Effectiveness studies in routine clinical settings | Literature review with undisclosed methodology | X | X | X |  |  |  |
| Andersson et al., 2019 | Peer-reviewed journal article | Internet-delivered EBPTs | Anxiety disorders + others | Not specified | Literature review with undisclosed methodology | X | X |  | X |  |  |
| Emmelkamp et al., 2020 | Peer-reviewed journal article | Virtual reality exposure therapy | Social anxiety disorder | Not specified | Literature review with undisclosed methodology |  | X |  |  |  |  |
| Erickson & Rector, 2021 | Peer-reviewed journal article | Assessment and CBT | Anxiety disorders in late life | Not specified | Literature review with undisclosed methodology | X |  | X |  | X |  |
| Gallo et al., 2013 | Peer-reviewed journal article | Barriers to access and direct-to-consumer marketing of EBPTs | Anxiety disorders | Overview of previous interventions relevant to the USA | Literature review with undisclosed methodology |  |  | X |  | X | X |
| Ishikawa et al., 2020 | Peer-reviewed journal article | Dissemination of CBT in Japan | Anxiety disorders + others | National healthcare system, Japan | Literature review with undisclosed methodology + historical perspective | X |  | X | X |  | X |
| Otto et al., 2012 | Peer-reviewed journal article | Brief CBT | Panic disorder | Not specified | Narrative review with undisclosed search methodology | X | X |  |  |  |  |
| Peachey et al., 2013 | Policy document | Models to increase access to psychological services | Mental health issues relevant to primary care services, including anxiety disorders | Mental healthcare across the nation, Canada | Literature review with undisclosed methodology |  |  |  | X |  | X |
| Richter et al., 2017 | Peer-reviewed journal article | Translating research to clinical care | CBT for anxiety disorders | Any routine care setting for CBT delivery | Literature review with undisclosed methodology | X | X | X |  |  |  |
| Weightman, 2020 | Peer-reviewed journal article | Digital psychotherapy, mainly iCBT | Anxiety disorders + depression | Rural and remote areas | Narrative review with unclear methodology | X | X | X |  |  | X |
| **Other documents (n = 20)** | | | | | | | | | | | |
| **Reference** | **Document type** | **Intervention** | **Clinical target** | **Setting** | **Data source** | **Types of strategies** | | | | | |
|  |  |  |  |  |  | **Contributing to the evidence base** | **Identifying CBT delivery modalities** | **Building capacity for CBT delivery** | **Attuning the process of access** | **Engaging potential service users** | **Improving programs and policies** |
| **Practice and/or policy guidelines (n = 4)** | | | | | | | | | | | |
| CADTH, 2019 | Policy document | Recommendations on implementing iCBT | Anxiety disorders + depression | National healthcare system, initial analysis for Ontario and generalized to the rest of Canada | Expert review of existing data + economic analysis + patient perspective |  | X | X |  |  | X |
| Health Quality Ontario, 2020 | Policy document | Providing high-quality care including access to CBT | Anxiety disorders | National healthcare system, ON, Canada | Existing evidence |  | X |  | X | X | X |
| NICE, 2011a | Policy document | Clinical and organizational practices for identification and referral to treatment | Common mental disorders (including anxiety disorders) | National healthcare system, England, UK | Expert review of previous guidelines + existing evidence | X |  |  | X |  |  |
| NICE, 2011b | Policy document | Recommendations for evidence-based management | Generalized anxiety disorder + panic disorder | National healthcare system, England, UK | Expert review of previous guidelines + existing evidence |  | X |  | X |  |  |
| **First-hand accounts from experts (n = 5)** | | | | | | | | | | | |
| Clark, 2011 | Peer-reviewed journal article | Improving access to EBPTs including CBT in the community | Anxiety disorders + depression | The IAPT national program, England, UK | Personal experience of the author + data relevant to the development and implementation of IAPT | X | X | X | X |  | X |
| Dugas, 2018 | Peer-reviewed journal article | Developing a more parsimonious treatment from mechanistic evidence | Generalized anxiety disorder | Series of experimental and observational studies, QC, Canada | Personal experience of the author + relevant publications throughout their career | X |  |  |  |  |  |
| Lindner et al., 2017 | Peer-reviewed journal article | Virtual reality exposure therapy | Specific phobias + social anxiety disorders + others | State-of-the-art regarding the development of virtual reality technology in 2016 | Personal experience of the authors + extent literature | X | X |  |  |  | X |
| Quero et al., 2015 | Peer-reviewed journal article | Exposure-based self-administered iCBT | Specific phobia (flying) | Program development at a university laboratory in Valencia, Spain | Personal experience of the authors + screenshots |  | X |  |  |  |  |
| Titov et al., 2018 | Peer-reviewed journal article | Implementation of iCBT in routine care | Comparative analysis for anxiety disorders + others | iCBT clinics in Sweden, Denmark, Norway, Canada and Australia | Personal experience of the authors + program data | X | X | X | X |  | X |
| **Commentaries (n = 2)** | | | | | | | | | | | |
| Hofmann, 2013 | Peer-reviewed journal article | Dissemination of CBT through improvement of the knowledge of practitioners | Anxiety disorders + others | Training in general, with some emphasis on issues relevant to the USA | Published articles + historical perspective | X |  | X |  |  | X |
| Taylor & Abramowitz, 2013 | Peer-reviewed journal article | Dissemination of evidence-based CBT | Anxiety disorders | Not specified | Published articles |  |  | X |  | X |  |
| **Opinion papers (n = 9)** | | | | | | | | | | | |
| Bentley et al., 2021 | Peer-reviewed journal article | Transdiagnostic CBT rationale and strategies | Anxiety disorders + depression | Not specified | Extent literature |  | X |  |  |  |  |
| Boeldt et al., 2019 | Peer-reviewed journal article | Virtual reality exposure therapy | Social anxiety disorder + panic disorder + phobias + others | Not specified | Extent literature | X | X | X |  | X |  |
| Cartreine et al., 2010 | Peer-reviewed journal article | Possible strategies to disseminate computer-based EBPTs | Anxiety disorders + others | Lays out a roadmap for the USA | Extent literature | X | X | X | X |  | X |
| Cougle, 2012 | Peer-reviewed journal article | Implementation of CBT | The role of parsimony, ease and efficiency for anxiety disorders + others | Not specified | Extent literature | X | X | X |  |  |  |
| Gaudiano & Ellenberg, 2014 | Peer-reviewed journal article | The evidence-based practice of psychotherapy (including CBT) | Anxiety disorders + others | Not specified | Extent literature |  | X | X |  | X | X |
| Gros et al., 2016 | Peer-reviewed journal article | Transdiagnostic psychotherapeutic practices (including CBT) | Anxiety disorders + others | Not specified | Extent literature |  | X |  |  |  |  |
| Gunter & Whittal, 2010 | Peer-reviewed journal article | Barriers to the dissemination of CBT | Anxiety disorders | Emphasis on translating published data to the context of North America | Extent literature | X | X | X | X |  | X |
| Harvey & Gumport, 2015 | Peer-reviewed journal article | Access to EBPTs | Modifiable barriers for various mental health issues | Emphasis on the USA | Extent literature | X | X | X | X | X | X |
| Khanna & Kendall, 2015 | Peer-reviewed journal article | Web-based training programs to improve competence in CBT | Anxiety disorders + others | Not specified | Extent literature |  |  | X |  |  |  |

**CBT:** cognitive-behavioral therapy **EBPT:** evidence-based psychological treatment **iCBT:** internet-delivered cognitive behavioral therapy
